# Nanorobot Hardware Architecture for Medical Defense

**DOI:** 10.3390/s8052932

**Published:** 2008-05-06

**Authors:** Adriano Cavalcanti, Bijan Shirinzadeh, Mingjun Zhang, Luiz C. Kretly

**Affiliations:** 1 CAN Center for Automation in Nanobiotech, Melbourne, VIC 3168 Australia; E-mail: adrianocavalcanti@canbiotechnems.com; 2 Robotics and Mechatronics Research Lab., Dept. of Mechanical Eng., Monash University, Clayton, Melbourne, VIC 3800 Australia; E-mail: bijan.shirinzadeh@eng.monash.edu.au; 3 Dept. of Mechanical, Aerospace & Biomedical Eng., The University of Tennessee, Knoxville, TN 37996 USA; E-mail: mjzhang@utk.edu; 4 Dept. of Microwave and Optics, Electrical & Comp. Eng., University of Campinas, Campinas, SP 13083 Brazil; E-mail: kretly@dmo.fee.unicamp.br

**Keywords:** Architecture, biohazard defense system, CMOS integrated circuits, device prototyping, hardware, medical nanorobotics, nanobioelectronics, nanobiosensor, proteomics

## Abstract

This work presents a new approach with details on the integrated platform and hardware architecture for nanorobots application in epidemic control, which should enable real time *in vivo* prognosis of biohazard infection. The recent developments in the field of nanoelectronics, with transducers progressively shrinking down to smaller sizes through nanotechnology and carbon nanotubes, are expected to result in innovative biomedical instrumentation possibilities, with new therapies and efficient diagnosis methodologies. The use of integrated systems, smart biosensors, and programmable nanodevices are advancing nanoelectronics, enabling the progressive research and development of molecular machines. It should provide high precision pervasive biomedical monitoring with real time data transmission. The use of nanobioelectronics as embedded systems is the natural pathway towards manufacturing methodology to achieve nanorobot applications out of laboratories sooner as possible. To demonstrate the practical application of medical nanorobotics, a 3D simulation based on clinical data addresses how to integrate communication with nanorobots using RFID, mobile phones, and satellites, applied to long distance ubiquitous surveillance and health monitoring for troops in conflict zones. Therefore, the current model can also be used to prevent and save a population against the case of some targeted epidemic disease.

## 1. Introduction

The development of nanorobots is a technological breakthrough that can enable real time *in vivo* prognosis for application in a variety of biomedical problems [1]. Particularly interesting is the fact that medical nanorobots should also provide an effective tool for defense against biohazard contaminants [2-4]. This paper presents the use of nanorobots with embedded protein based nanobiosensors [5], providing a practical molecular machine for medical defense technology.

Normally, for areas in public calamity or conflict zones, the absence of drinking water, any sort of fuel, electricity, and the lack of towers for network communication, including cable and wireless telephony, is a constant [6]. In such a situation, the available infrastructure is far from ideal to enable a large scale medical laboratory with precise and fast analysis. For such aspect, nanorobots integrated with nanobiosensors can help to transmit real time information, using international mobile phones for wireless data transmission through satellite communication [5,7,8]. In fact, nanorobots should mean an efficient and powerful clinical device to provide precious biomedical monitoring [9], both for soldiers as for civilian population. Therefore, the architecture presented in this work can help to address the development of just in time accurate information, protecting lives in urban areas against biohazard materials.

The proposed hardware architecture aims the use of medical nanorobots as an integrated platform to control contagious epidemic diseases [1,10]. Details on communication required for surveillance assistance, and the integration platform to interface long distance monitoring with nanorobots are also given through the paper. Thus, the present model serves to help monitoring contagious diseases [11], which in practical ways should protect personnel on patrol across conflict areas or during humanitarian missions. Furthermore, an important and interesting aspect in the proposed architecture is the fact that the same technique can be useful for other situations, like natural catastrophes or possible biohazard contamination [12], helping against pandemic outbreaks [13], when time and fast information is a key factor for public management [14,15].

To visualize how stages of the actual and in development technologies can be used to biohazard defense, the nanorobots are applied to detect influenza inside body based on blood flow patterns and protein signals [16,17]. The nanorobot architecture and integrated system are described [1,5,18], and the nanobiosensor is simulated based on electrochemical properties for digital-analog sensor activation. Therefore, the work developed is also useful as a practical methodology for control and equipment design analyses.

## 2. Nanorobot Development for Defense

The defense industry should remarkably benefit from achievements and trends on current nanobiotechnology systems integration. Such trends on technology have also resulted in a recent growing interest from the international scientific community, including medical and pharmaceutical sectors, towards the research and development of molecular machines.

### 2.1. Medical Nanorobots

The research and development of nanorobots with embedded nanobiosensors and actuators is considered a new possibility to provide new medical devices for doctors [9,19-21]. As integrated control mechanisms at microscopic environments differ from conventional control techniques, approaches using event-based feed forward control are sought to effectively advance new medical technologies [22,23]. In the same way the development of microelectronics in the 1980s has led to new tools for biomedical instrumentation, the manufacturing of nanoelectronics [24,25], will similarly permit further miniaturization towards integrated medical systems, providing efficient methodologies for pathological prognosis [26-28].

The use of microdevices in surgery and medical treatments is a reality which has brought many improvements in clinical procedures in recent years [29]. For example, among other biomedical instrumentation, catheterization has been successfully used as an important methodology for heart and intracranial surgery [30-32]. Now the advent of biomolecular science and new manufacturing techniques is helping to advance the miniaturization of devices from micro to nanoelectronics. Sensors for biomedical applications are advancing through teleoperated surgery and pervasive medicine [33-35], and this same technology provides the basis for manufacturing biomolecular actuators. A first series of nanotechnology prototypes for molecular machines are being investigated in different ways [18,36-38], and some interesting devices for propulsion and sensing have been presented [39-41]. More complex molecular machines, or nanorobots, having embedded nanoscopic features represent new tools for medical procedures [42-44].

### 2.2. Motivation

Worldwide infectious and microbial diseases account for approximately 40% of the total 50 million annual deaths [45]. Considering the contagious properties of biohazard materials, they mean a serious threat that can affect a whole population, especially for metropolitan areas, where a contamination can spread extremely fast. Dealing with such a problem, time is a major issue. Although traditional methods for clinical analysis of contamination is useful to positively identify if a person was infected with some sort of virus, this laboratorial process demands a precious time and a complex infrastructure. However, for conflict zones such infrastructure is often not easily accessible.

Taking from the moment of infection, some contagious diseases may show the first symptoms after hours, a week, or longer time, like years or even decades [12,46]. It means, for example, that when the public authorities noticed the infection from a contaminated person, showing external symptoms, a virus had enough time to spread itself through a circle of friends and workmates of the infected victim. Meantime, those mates were adversely driving the virus forward, and had started a catastrophic chain circle [13]. The use of nanorobots with embedded nanodevices for real time epidemic control, as lab on a chip, can be useful to avoid serious contamination with large proportions. In fact, it can help save a large part of a population in terms of fast evacuation and effective patients' quarantine. Thus, it should enable a more effective action against biohazard materials.

We implemented a system simulation and architecture of nanorobots for sensing the bloodstream, targeting biochemical changes against pathological signals. Actual advances in wireless technologies, nanoelectronics devices, and their use in the implementation of nanorobots applied to epidemic control, illustrate what upcoming technologies can enable in terms of real time health monitoring.

The approach for *in vivo* monitoring chemical concentrations should also apply to other biomedical problems, and likewise be useful for prognosis of complex diseases and phamacokinetics control. Furthermore, in the proposed platform architecture, different programs and commands can be sent and information retrieved from inside body through wireless communication, providing important aspects on interface and medical instrumentation of nanorobots.

### 2.3. Prevention and Control

The World Health Organization (WHO) has started in 1948 the initiative to implement a worldwide identification of new influenza viruses [14]. Currently demand for vaccines and effective ways to quickly manage and fight a pandemic outbreak are enormous, which also motivated WHO to develop the Global Outbreak Alert and Response Network, enhancing the world's collaboration in containment of infectious diseases [47].

Some highly contagious germs, such as SARS (severe acute respiratory syndrome) [15], smallpox [11] and influenza [17], can bring deadly consequences, and spread easily across borders and among populations from different countries. In face of international security demand for defense against new threats driven by possibly biohazard outbreaks, the current $13 billion global vaccine business should grow 18% a year to $30 billion by 2011 [48]. The concern to avoid personnel losses has also motivated the implementation of periodical crew immunization of US Navy against influenza and other plagues as surveillance safety action [2,3]. The concern in this matter, in order to save and protect lives, help us to understand how important is to improve population-wide disease outbreak detection [49], preventing any pandemic onset. In fact, a pandemic influenza outbreak would likely cause the most severe vaccine shortages to date with global consequences [50].

Notwithstanding that improved drugs and vaccines have evolved a lot, antimicrobials are of limited usefulness due to the following aspects: antimicrobial resistance to drugs and antibiotics, the large number of possible microbes that can be used for weapons, and limitations in technical feasibility for developing vaccines and effective antibacterials against certain germs [3,4]. Therefore, in recent years a crescent concern and interest has emerged for methods to efficiently protect people lives not only through immunization, but also and even more accurately through advanced real time biomolecular *in vivo* virus detection [51].

An efficient bioharzard defense system should address frequent collection of data, fast information transfer, early signature of the outbreak, immediate analysis of incoming data, and immediate output [10]. On such aspects, the current trends on new nanobiosensors, and miniaturization of micro to nanoelectronics, open new possibilities with the development of medical nanorobotics for the implementation of efficient biohazard defense systems.

## 3. Influenza Characteristics

Time for incubation of pandemic syndromes may vary from one contagious plague to another, and first symptoms can be predicted given clinical information and previous historic occurrences, using statistical models. The size of an outbreak is directly correlated and influenced by the delay for recognition about the contaminated area. The incubation period of disease is the time from exposure to the infectious agent to the onset of disease, and depending on the infection dose of influenza, it can vary about 2 to 5 days [13]. For influenza, the contamination can happens through inhalation, ingestion, or direct contact through hand shaking and conversation. Influenza can live in ducks, chickens, wild birds, horses, pigs and humans.

**Figure 1.**
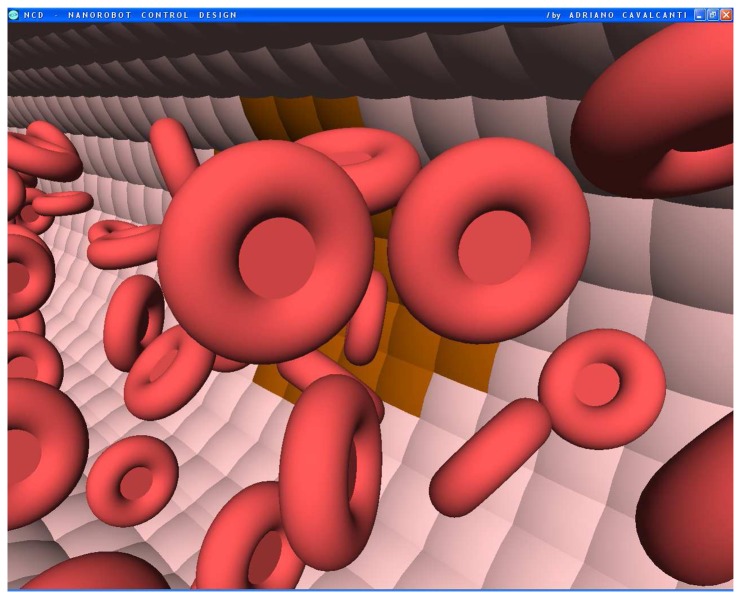
The bloodstream flows through the vessel in the 3D model. The vessel endothelial cells denote in brown color the influenza virus beginning to spread from one cell to another.

The influenza virus invades cell (Fig. 1), and after the cell invasion, it makes use of enzymes to decrease intracellular pH, slightly increasing ∼1°C intracellular temperature, which is used to accelerate virus cell fusion activity [52-54]. Before a person shows symptomatic reactions, short after being infected by influenza, the bloodstream begins to receive a higher concentration of alpha-N-acetylgalactosaminidase (alpha-NAGA), which is secreted from the invaded cells [16]. The protein hemagglutinin serves as virus envelope for influenza, promoting alpha-NAGA signals. Alpha-NAGA is a protein identified through the genome mapping, which belongs to chromosome 22 [55]. The lack of macrophage, incurred from the alpha-NAGA enzyme secreted through the infected cells, leads to immunosuppression and helps the virus to spread easily through the body.

Thus, this change of chemical concentration, with overexpression of alpha-NAGA in the bloodstream, is used to trigger the nanorobot prognostic behavior, which sends electromagnetic backpropagated signals to the mobile phone carried with the person. As an integrated biohazard defense system, once the nanorobot activated the cell phone, this information is retransmitted for the satellites utilized as feasible telecommunication system. Whenever the central is alarmed about the *case zero*, the administration takes the necessary action, automatically sending SMS (short message service) for the near troop members, inside an area with a radius of approximately 20KMs, informing identification and the current position of the person who is contaminated. Technically, the *case zero* is the first occurrence of someone contaminated by the influenza in certain area, which means that a pandemic is running anywhere else close to that location.

## 4. Nanobioelectronics

Current developments in nanoelectronics [56] and nanobiotechnology [57] are providing feasible development pathways to enable molecular machine manufacturing, including embedded and integrated devices, which can comprise the main sensing, actuation, data transmission, remote control uploading, and coupling power supply subsystems, addressing the basics for operation of medical nanorobots.

A recent actuator with biologically-based components has been proposed [58]. This actuator has a mobile member that moves substantially linearly as a result of a biomolecular interaction between biologically-based components within the actuator. Such actuators can be utilized in nanoscale mechanical devices to pump fluids, open and close valves, or to provide translational movement.

To help control nanorobot position, a system for tracking an object in space can comprise a transponder device connectable to the object. The transponder device has one or several transponder antennas through which a transponder circuit receives an RF (radio frequency) signal. The transponder device adds a known delay to the RF signal, thereby producing RF response for transmitting through the transponder antenna [59]. A series of several transmitters and antennas allow a position calculator, associated with the transmitters and receivers, to calculate the position of the object as a function of the known delay, and the time period between the emission of the RF signal and the reception of the RF response from the first, second and third antennas.

Nanotechnology is moving fast towards nanoelectronics fabrication. Chemically assembled electronic nanotechnology provides an alternative to using complementary metal oxide semiconductor (CMOS) for constructing circuits with feature sizes in the tens of nanometers [60]. A CMOS component can be configured in a semiconductor substrate as part of the circuit assembly [24]. An insulating layer is configured on the semiconductor substrate, which covers the CMOS component. A nanoelectronic component can be configured above an insulating layer. If several nanoelectronic components are provided, they are preferably grouped in nanocircuit blocks [24].

Biosensors are currently used to incorporate living components, including tissues or cells which are electrically excitable or are capable of differentiating into electrically excitable cells, and which can be used to monitor the presence or level of a molecule in a physiological fluid [61]. CNTs (carbon nanotubes) and DNA (deoxyribonucleic acid) are recent candidates for new forms of nanoelectronics [62]. These are combined to create new genetically programmed self-assembling materials for facilitating the selective placement of CNTs on a substrate by functionalizing CNTs with DNA. Through recombinant DNA technology, targets labeled with distinct detectable biomarkers can be defined, such as fluorescent labels, enzyme labels, or radioactive patterns, and employed as suitable protein transducers [63].

## 5. Integrated System Platform

The proposed model uses electromagnetic radio waves to command and detect the current status of nanorobots inside the body. Therefore, the cell phone is applied for medical nanorobotics platform [7,64,65]. This occurs as the cell phone emits a magnetic signature to the passive CMOS sensors embedded in the nanorobot, which enables sending and receiving data through electromagnetic fields [66]. From the last set of events recorded in pattern arrays, information can be reflected back by wave resonance [64].

The nanorobot model includes embedded IC (integrated circuit) nanoelectronics [67], and the architecture involves the use of satellites and mobile phones for data transmission and coupling energy [68,69]. The nanorobot is programmed for sensing and to detect concentration of alpha-NAGA in the bloodstream [7,16,70]. The nanorobot architecture uses an RFID (radio frequency identification device) CMOS transponder system for *in vivo* positioning [70], adopting well established communication protocols, which allow track information about the nanorobot position [5,7].

The ability to manufacture nanorobots should result from current trends and new methodologies in fabrication, computation, transducers and nanomanipulation. Depending on the case, different gradients on temperature, concentration of chemicals in the bloodstream, and electromagnetic signature are some of relevant aspects when monitoring *in vivo* biochemical parameters. CMOS VLSI (very-large-scale integration) design using deep ultraviolet lithography provides high precision and a commercial way for manufacturing early nanodevices and nanoelectronics systems. Innovative CMOSFET (complementary metal oxide semiconductor field effect transistor) and some hybrid techniques should successfully drive the pathway for the assembly processes needed to manufacture nanorobots, where the joint use of nanophotonics and CNTs can even accelerate further the actual levels of resolution ranging from 248nm to 157nm devices [71]. To validate designs and to achieve a successful implementation, the use of VHDL (very high speed integrated circuit hardware description language) has become the most common methodology utilized in the integrated circuit manufacturing industry [72].

## 6. Nanorobot Architecture

**Figure 2.**
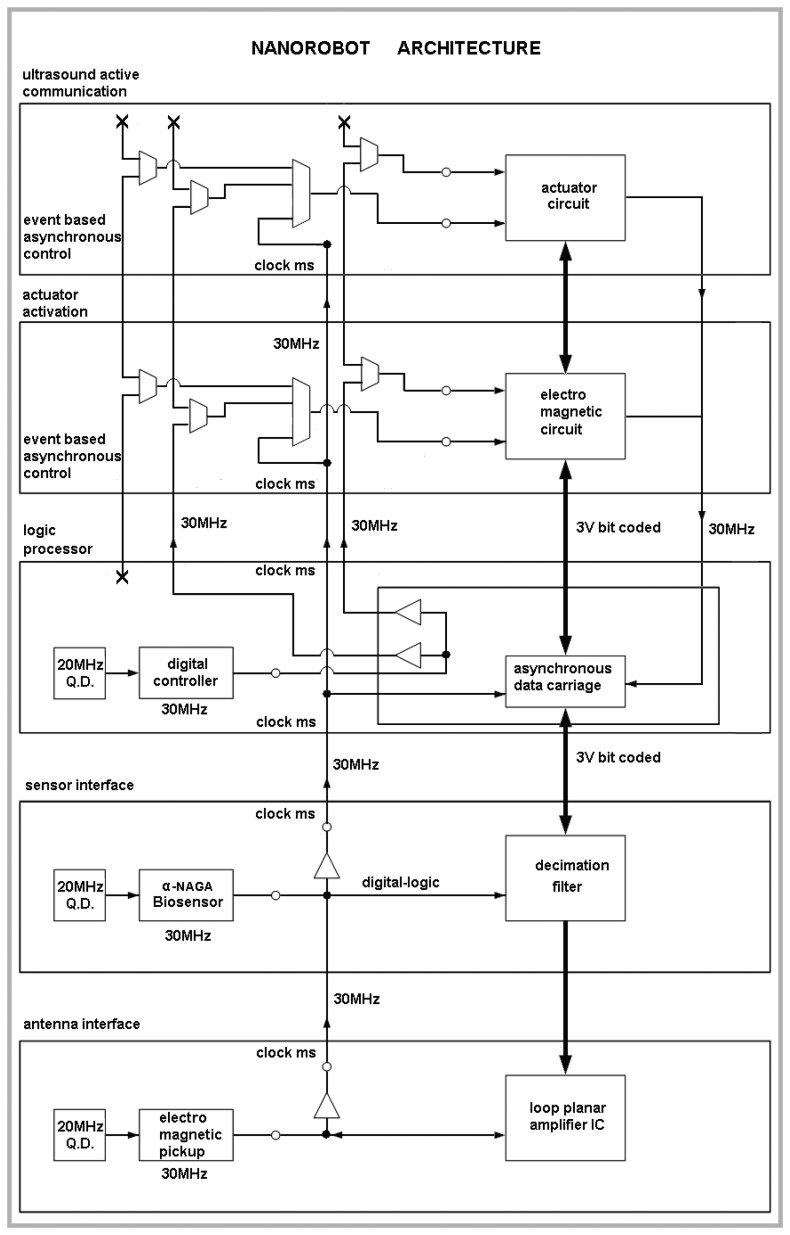
Integrated circuit block diagram.

The medical nanorobot for biohazard defense should comprise a set of integrated circuit block as an ASIC (application-specific integrated circuit). The architecture has to address functionality for common medical applications [18], providing asynchronous interface for antenna, sensor, and a logic nanoprocessor, which is able to deliberate actuator and ultrasound communication activation when appropriate (Fig. 2). The main parameters used for the nanorobot architecture and its control activation, as well as the required technology background that can advance manufacturing hardware for molecular machines, are described next. As a practical rule, the number of nanodevices to integrate a nanorobot should keep the hardware sizes in regard to inside body operation applicability.

### 6.1. Chemical Sensor

Manufacturing silicon-based chemical and motion-sensor arrays using a two-level system architecture hierarchy has been successfully conducted in the last 15 years. Applications range from automotive and chemical industry, with detection of air to water element pattern recognition, through embedded software programming, and biomedical analysis. Through the use of nanowires, existing significant costs of energy demand for data transfer and circuit operation can be decreased by up to 60% [67]. CMOS-based sensors using nanowires as material for circuit assembly can achieve maximal efficiency for applications regarding chemical changes, enabling new medical applications [21,73,74].

Sensors with suspended arrays of nanowires assembled into silicon circuits, decrease drastically self-heating and thermal coupling for CMOS functionality [75]. Factors like low energy consumption and high-sensitivity are among some of the advantages of nanosensors [76]. Nanosensor manufacturing array processes can use electrofluidic alignment to achieve integrated CMOS circuit assembly as multielement systems [67]. Passive and buried electrodes can be used to enable cross-section drive transistors for signal processing circuitry readout. The passive and buried aligned electrodes must be electrically isolated to avoid loss of processed signals. For the nanorobot architecture, the antibody anti-digoxigenin is included for modelling the IC biosensor; the antibody serves to identify higher concentrations of proteins that couple alpha-NAGA isoforms to intracellular bloodstream signaling [45]. The nanobiosensor provides an efficient integrated way for nanorobots identifying the locations with occurrences of alpha-NAGA. Enzyme secretion from cell hostage produces alpha-NAGA overexpression, which is denoted by changes of gradients in the bloodstream. Therefore, an efficient prognostic can be achieved, even before symptomatic reactions, helping to fight a virus outbreak. Carbon nanotubes serve as ideal materials for the basis of a CMOS IC nanobiosensor [67,77,78].

Some limitations to improving BiCMOS (bipolar-CMOS), CMOS and MOSFET methodologies include quantum-mechanical tunneling for operation of thin oxide gates, and subthreshold slope. However, the semiconductor branch has moved forward to keep circuit capabilities advancing. Smaller channel length and lower voltage circuitry for higher performance are being achieved with biomaterials aimed to attend the growing demand for high complex VLSIs. New materials such as strained channel with relaxed SiGe (silicon-germanium) layer can reduce self-heating and improve performance [79]. Recent developments in three-dimensional (3D) circuits and FinFETs double-gates have achieved astonishing results and according to the semiconductor roadmap should improve even more. To further advance manufacturing techniques, silicon-on-insulator (SOI) technology has been used to assemble high-performance logic sub 90nm circuits [80]. Circuit design approaches to solve problems with bipolar effect and hysteretic variations, based on SOI structures, have been demonstrated successfully [79]. Thus, while 10nm circuits are currently under development, already-feasible 45nm NanoCMOS ICs represent breakthrough technology devices that are currently being utilized in products.

### 6.2. Actuator

There are different kinds of actuators, such as electromagnetic, piezoelectric, electrostatic, and electrothermal. Which can be utilized, depending the aim and the workspaces where it will be applied [81]. Flagella motor has been quoted quite frequently as an example for a kind of biologically inspired actuator for molecular machine propulsion [82]. Adenosine triphosphate, also know for short as ATP, is equally used as an alternative for nanomotors [83]. DNA and RNA (ribonucleic acid) prototypes were also proposed for designing different types of devices.

A set of fullerene structures were presented for nanoactuators [84]. The use of CNTs as conductive structures permits electrostatically driven motions providing forces necessary for nanomanipulation. CNTs can be used as materials for commercial applications on building devices and nanoelectronics such as nanotweezers and memory systems. SOI technology has been used for transistors with high performance, low heating and low energy consumption for VLSI devices. CNT selfassembly and SOI properties can be combined to addressing CMOS high performance on design and manufacturing nanoelectronics and nanoactuators [85]. Owing to the maturity of silicon CMOS technology, as well as the unique properties of CNTs, the integration of CNT and the CMOS technology can make use of the advantages of both [86].

For a medical nanorobot, applying CMOS as an actuator based on biological patterns and CNTs is proposed for the nanorobot architecture as a natural choice. In the same way DNA can be used for coupling energy transfer, and proteins serve as basis for ionic flux with electrical discharge ranges from 50-70 mV dc voltage gradients in cell membrane [87], an array format based on CNTs and CMOS techniques could be used to achieve nanomanipulators as an embedded system for integrating nanodevices of molecular machines [56]. Ion channels can interface electrochemical signals using sodium for the energy generation which is necessary for mechanical actuators operation [87]. Embedded actuators are programmed to perform different manipulations, enabling the nanorobot a direct active interaction with the bloodstream patterns and molecular parameters inside the body.

### 6.3. Power Supply

The use of CMOS for active telemetry and power supply is the most effective and secure way to ensure energy as long as necessary to keep the nanorobot in operation. The same technique is also appropriate for other purposes like digital bit encoded data transfer from inside a human body [88]. Thus, nanocircuits with resonant electric properties can operate as a chip, providing electromagnetic energy supplying 1.7 mAat 3.3V for power, which allows the operation of many tasks with few or no significant losses during transmission [65]. RF-based telemetry procedures have demonstrated good results in patient monitoring and power transmission through inductive coupling [66], using well established techniques already widely used in commercial applications of RFID. The energy received can also be saved in ranges of ∼1μW while the nanorobot stays in inactive modes, just becoming active when signal patterns require it to do so. Some typical nanorobotic tasks may require the device only to spend low power amounts, once it has been strategically activated. For communication, sending RF signals ∼1mW is required. Allied with the power source devices, the nanorobots need to perform precisely defined actions in the workspace, using available energy resources as efficiently as possible.

A practical way to achieve easy implementation of this architecture should obtain both energy and data transfer capabilities for nanorobots by employing cell phones in such process [7]. The mobile phone can be uploaded with the control software that includes the communication and energy transfer protocols.

### 6.4. Data Transmission

The application of devices and sensors implanted inside the human body to transmit data for a person health care can enable great advantages in continuous medical monitoring [89]. It can also provides an innovative tool for accurate and in time prognosis of contagious diseases. Most recently, the use of RFID for *in vivo* data collecting and transmission was successfully tested for electroencephalograms [65]. For communication in liquid workspaces, depending on the application, acoustic, light, RF, and chemical signals may be considered as possible choices for communication and data transmission. Chemical sensing and signaling can be quite useful for nearby orientation and communication purposes among nanorobots [1,90]. Acoustic communication is more appropriate for longer distance communication and detection with low energy consumption as compared to light communication approaches [5,91]. Although optical communication permits faster rates of data transmission, its energy demand makes it not ideal for medical nanorobotics [92].

Works with RFID have been developed as an integrated circuit device for medicine [70]. Using integrated sensors for data transfer is the better answer to read and write data in implanted devices. Thus, the nanorobot should be equipped with single-chip RFID CMOS based sensors [65,93]. CMOS with submicron SoC design addresses extremely low power consumption for nanorobots communicating collectively at longer distances through acoustic sensors. For communication, as well as for navigational purposes, the use of nanoacoustics for nanorobot interactions can effectively achieve resolutions of 700nm [94]. For data recognition, the acoustic phonons scattered from the origin should be propagated at sufficient distances, and the acoustic wavefield should be measured by diffraction propagation. For the nanorobot active sonar communication, frequencies can reach up to 20μW@8Hz at resonance rates with 3V supply [91].

More widely accepted and usual than an RF CMOS transponder, mobile phones can be extremely practical and useful as sensors for acquiring wireless data transmission from medical nanorobots implanted inside the patient's body. Cell phones can be a good choice for monitoring predefined patterns in various biomedical applications, such as helping in ubiquitous health care for real time influenza detection. To accomplish that, chemical nanobiosensors should be embedded in the nanorobot to monitor alpha-NAGA levels. The nanorobot emits signals to send an alarm, in case of detection of any alpha-NAGA protein overexpression, denoting when a person was contaminated with influenza. For nanorobot passive data transferring ∼4.5 kHz frequency with approximate 22μs delays are possible ranges for data communication.

In our molecular machine architecture, to successfully set an embedded antenna with 200nm size for the nanorobot RF communication, a small loop planar device is adopted as an electromagnetic pick-up having a good matching on low noise amplifier (LNA); it is based on gold nanocrystal with 1.4nm^3^, CMOS and nanoelectronic circuit technologies [65,95]. Frequencies ranging from 1 to 20MHz can be successfully used for biomedical applications without any damage [65].

## 7. System Implementation

The nanorobot model prototyping uses a task based approach with detection of protein alpha-NAGA higher concentrations. The simulation and analysis consist of adopting a multi-scale view of the scenario with bloodstream simulation. It incorporates the physical morphology of the biological environment along with physiological fluid flow patterns, and this is allied with the nanorobot systems for orientation, drive mechanisms, sensing and control. The real time 3D simulation is used to achieve high-fidelity on control modelling and equipment prototyping. Hence, the NCD (Nanorobot Control Design) software was implemented and is used for nanorobot sensing and actuation. The computational model is applied as a practical tool for control and manufacturing design analyses. Real time 3D design and simulation are important for the fast development of nanotechnology, helping also in the research and development of medical nanorobots [96,97]. Such tools have significantly supported the semiconductor industry to achieve faster VLSI implementation [98]. It has similarly direct impact on nanomanufacturing and also nanoelectronics progress [24]. Simulation can anticipate performance, help in new device prototyping and manufacturing, nanomechatronics control design and hardware implementation [1,80].

**Figure 3.**
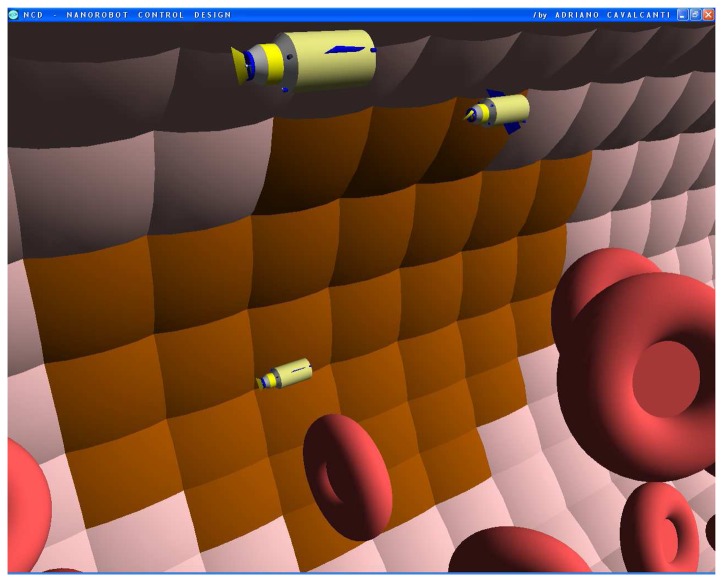
Infected cells in brown color represented as early stage of virus cell invasion.

**Figures 4.**
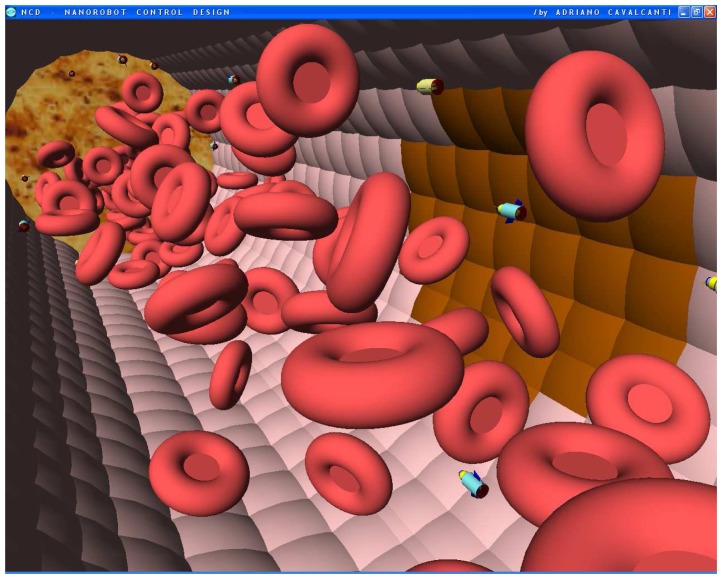
Screenshots with nanorobots and red blood cells inside the vessel. The real time 3D simulation optionally provides visualization either with or without the red blood cells. The influenza infection with cell hostage begins to spread from infected to nearby uninfected cells. The nanorobots flow with the bloodstream sensing for protein overexpression.

The nanorobot exterior shape being comprised of carbon-metal nanocomposites [99], to which should be attached an artificial glycocalyx surface [100], is used to minimize fibrinogen and other blood proteins adsorption or bioactivity, ensuring sufficient biocompatibility to avoid immune system attack [63,92]. Different molecule types are distinguished by a series of chemotactic sensors whose binding sites have a different affinity for each kind of molecule [42,101]. These sensors can also detect obstacles which might require new trajectory planning [102]. The nanorobot sensory capabilities are simulated, allowing it to detect and identify the nearby possible obstacles in its environment, as well as alpha-NAGA protein overexpression for continuous real time *in vivo* prognosis purpose. For chemical detection a variety of sensors is possible, enabling identification of various types of cells [42,61,67,75]. A set of different views from the 3D environment can be observed (Figs. 3 and 4). A multiplicity of nanorobots allows precise detection of alpha-NAGA in initial stages of influenza infection.

## 8. Physical Parameters

The microenvironments of the circulatory system vary considerably in size, flow rates, and other physical properties. Chemicals in the blood can present distinct diffusion coefficients, and like in any other surgical, prognosis, or integrated pharmacokinetic system, there is a range of plausible designs for the nanorobots depending on customized requirements [5]. In defining the nanorobot application, physical parameters is the key point to determining the architecture prototype [1], sensor based actuation [102], and strategies to increase the medical instrumentation efficiency [18].

**Figures 5.**
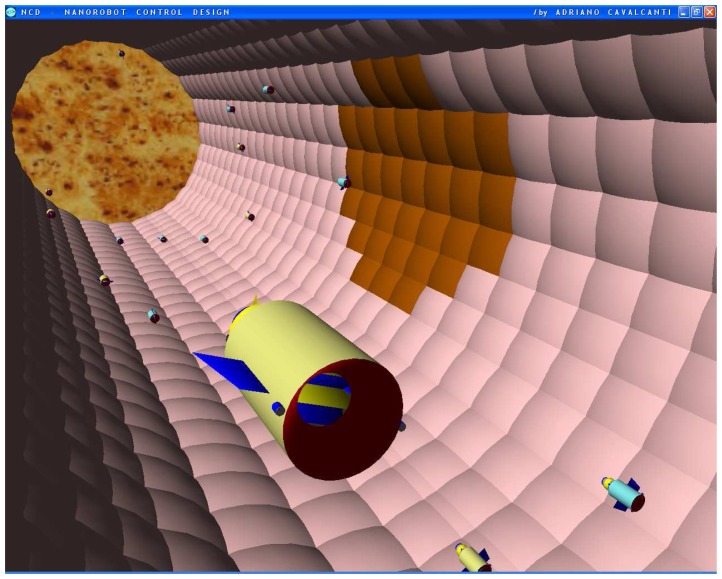
Screenshots with nanorobots and red blood cells inside the vessel. The real time 3D simulation optionally provides visualization either with or without the red blood cells. The influenza infection with cell hostage begins to spread from infected to nearby uninfected cells. The nanorobots flow with the bloodstream sensing for protein overexpression.

Small vessels have diameters of up to several tens of microns, and lengths of about a millimeter. Notwithstanding our control actuation can be set with different parameters, such as adjusting detection thresholds, we adopted typical values for these properties. The workspace used in the simulator comprised an environment consisting of a segment of the vessel with length *L* = 60*μm* and diameter *K* = 30*μm*. The model has also a small group of hostage cells, as the medical target on the vessel wall (Fig. 5), releasing alpha-NAGA proteins into the fluid. Cells and nanorobots continually enter one end of the workspace along with the fluid flow. We treat nanorobots not responding while within the workspace as if they did not detect any signal, so they flow with the fluid as it leaves the workspace. Thus, we choose the workspace length sufficient to include the region where the chemical from the target is significantly above the background level. The cells occupy about 1/5-th of the workspace volume, a typical *hematocrit* value for small blood vessels.

The nanorobot morphology is based on microbiology, presenting a cylinder's shape with 2*μm* in length and 0.5*μm* in diameter, which allows free operation inside the body [103]. Therefore, the nanorobot's customized design is useful for health monitoring, but it also enables the nanorobot to cross the blood brain barrier for other biomedical applications, such as required for intracranial therapies. This prototyping allows the nanorobot to have a complete kinematic motion control in regard to Brownian motion events inside microenvironments at low Reynolds number.

The simulator comprises a real time 3D environment, including nanorobots and chemical signal parameters. Most of the cells are red blood cells, with 6*μm* diameter. The number densities of platelets and white blood cells are about 1/20-th and 1/1000-th that of the red cells, respectively. As specific example, we consider alpha-NAGA protein signal, produced in response to the influenza, having molecular weight of 52 kDa (kilodaltons), with concentration near the hostage cells at ≈*30ng*/*ml* and background concentration in the bloodstream about 300 times smaller. This choice provides an interesting nanorobot task, though we could equally well study tasks involving chemicals with different concentrations relevant for other similar biomedical problems, such as for new drug target to fight HCV (hepatitis C virus) or HIV (human immunodeficiency virus) [1,16,46,90]. In our study, the chemical signal was taken to be produced uniformly throughout vessel once the person was infected by influenza at the rate Q. This rate changes in proportion to the disease progression.

## 9. Target Identification

**Figures 6.**
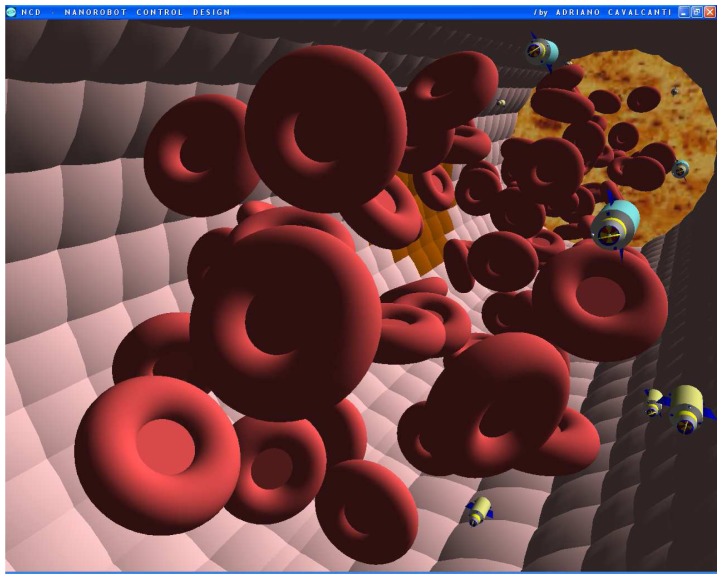
Screenshots with nanorobots and red blood cells inside the vessel. The real time 3D simulation optionally provides visualization either with or without the red blood cells. The influenza infection with cell hostage begins to spread from infected to nearby uninfected cells. The nanorobots flow with the bloodstream sensing for protein overexpression.

**Figures 7.**
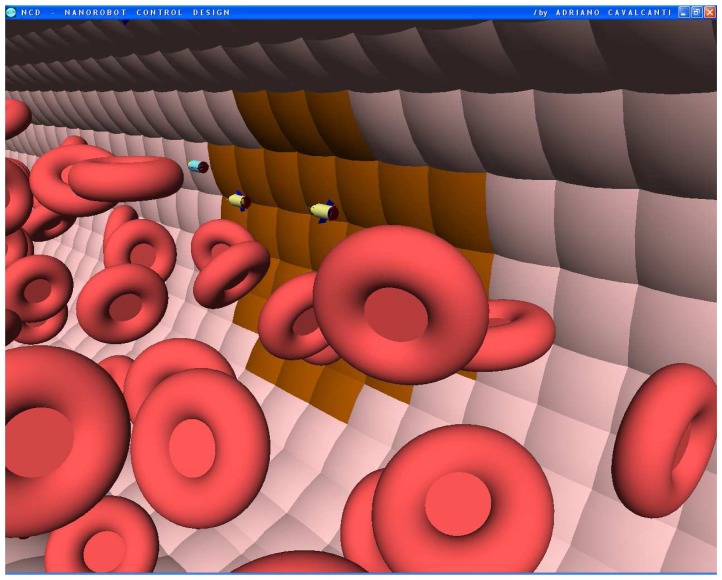
Screenshots with nanorobots and red blood cells inside the vessel. The real time 3D simulation optionally provides visualization either with or without the red blood cells. The influenza infection with cell hostage begins to spread from infected to nearby uninfected cells. The nanorobots flow with the bloodstream sensing for protein overexpression.

Nanorobots using chemical sensors as embedded nanoelectronics can be programmed to detect different levels of alpha-NAGA signals. Based on clinical analysis, the alpha-NAGA proteins are well established as medical targets for early stages of influenza development [16]. Nanorobots as mobile medical devices injected through the bloodstream are used in our study; the medical 3D environment comprises historical clinical data of blood flow patterns and morphological parameters from patients with influenza virus (Figs. 6 and 7). The behaviour used by influenza to cell invasion and fusion is quite similar with tactics also used by other viruses, like Smallpox or SARS. The proposed platform with nanorobot prototype as a quite effective architecture applied to influenza prognosis, can also address a broad range of biohazard defense possibilities, therefore providing a new virus fighting technology.

Based on precise personnel health monitoring, the presented model can support the military command headquarters towards a pervasive surveillance integrated platform for medical defense. The nanorobot computation is performed through asynchronous integrated circuit architecture with a task based modular approach. The embedded nanobiosensor is used for detection of alpha-NAGA concentrations in the bloodstream. Due to background compounds, some detection occurs even without alpha-NAGA concentrations specified as influenza infection. Therefore, for the chemical diffusion a capture rate α is adopted for influenza identification, given the radius *R* for a region with concentration as:

(1) α=4πDRC.

*D* represents the diffusion coefficient, and *C* is the chemical concentration [104]. With independent random motions for the molecules, detection over a time interval Δ*t* is based on a Poisson process with mean value αΔ*t*. When objects occupy only a small fraction of the volume, the velocity at distance *r* from the center of the vessel is represented by:

(2) $$w=2\nu \left(1-{\left(r/\left(d/2\right)\right)}^{2}\right).$$

The velocity has a parabolic flow in relation to the cells. For a fluid moving at velocity *v* in the positive *x*-direction, it passing a plane containing a point of a chemical source produced at a rate *Q* (molecules per second), and a diffusion coefficient *D*. Thus, diffusion equation is defined as:

(3) $$D\nabla^{2}C=\nu \partial C/\partial x.$$

The boundary conditions attain a steady point source at the origin, having no net flux across the boundary plane at *y* = 0; thereby the steady-state concentration C (molecules per *μm*^3^) is determined at point (*x*, *y*, *z*) by [1]:

(4) $$C\left(x,y,z\right)=\frac{Q}{2\pi Dr}e^{-\nu \left(r-x\right)/\left(2D\right)}$$

and *r* is the distance to the source:

(5) $$r=\sqrt{x^{2}+y^{2}+z^{2}}.$$

## 10. Nanorobot Simulation and Results

A range of different pattern signals are directly correlated to specific diseases. Hence, chemical signals can serve for medical target identification, diagnosis, and actuation [1]. For the problem of a pandemic virus, the nanorobots are used for identifying and to predict bloodstream protein parameters, which can prevent against chemical reactivity hazards. A set of proteins or specific self-assembled chemical cells can be characterized as a typical virus, with profound consequences for a large population in the case of epidemic proliferation.

Nanobioelectronics, using nanowires as material for embedded biosensors and integrated circuit packaging, can achieve maximal efficiency for applications regarding chemical changes [5]. Thus, using chemical sensors, nanorobots can be programmed to detect different levels of distinct proteins. The nanorobot should be useful, therefore, to find a virus, which may be proliferating into a person's bloodstream through cell invasion. Integrated nanobiosensors can be utilized enabling precise cell biology interfaces, and detecting different concentrations of chemical signals inside the body, it should provide real time medical monitoring to fight an epidemic disease in initial stages of contamination [12,105,106].

**Figures 8.**
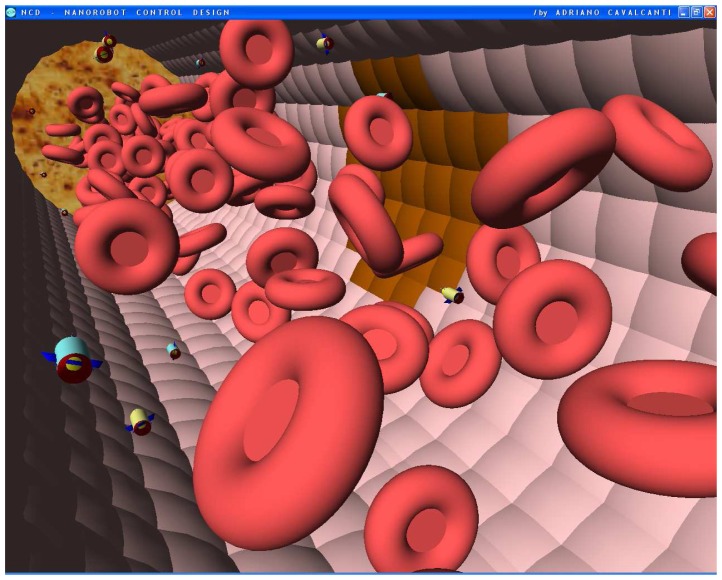
Screenshots with nanorobots and red blood cells inside the vessel. The real time 3D simulation optionally provides visualization either with or without the red blood cells. The influenza infection with cell hostage begins to spread from infected to nearby uninfected cells. The nanorobots flow with the bloodstream sensing for protein overexpression.

**Figures 9.**
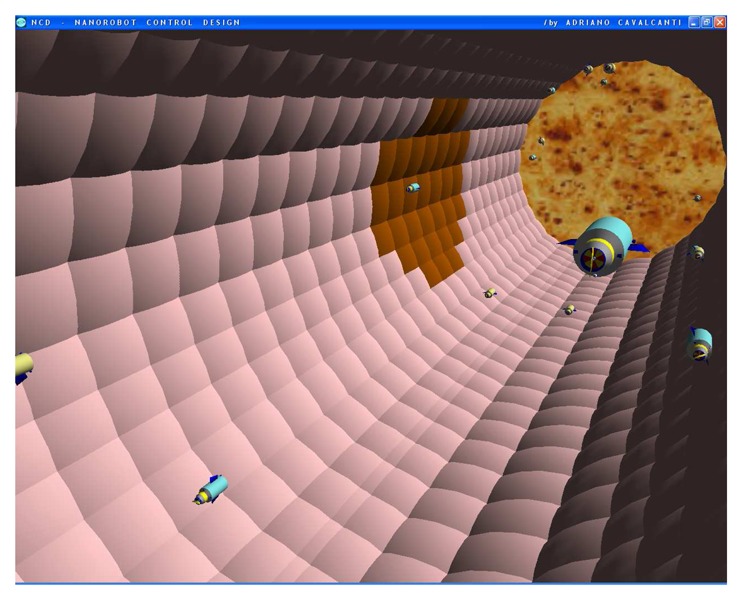
Screenshots with nanorobots and red blood cells inside the vessel. The real time 3D simulation optionally provides visualization either with or without the red blood cells. The influenza infection with cell hostage begins to spread from infected to nearby uninfected cells. The nanorobots flow with the bloodstream sensing for protein overexpression.

The chemical detection in a complex dynamic environment is an important factor to consider for nanorobots in the task of interacting with the human body. The nanorobots need to track the influenza development before a pandemic outbreak happens. The main cell morphological changes, given influenza infection, were taken for modelling bloodstream, which provides the necessary environment for medical nanorobot interaction analysis and prototyping (Figs. 8 and 9).

**Figure 10.**
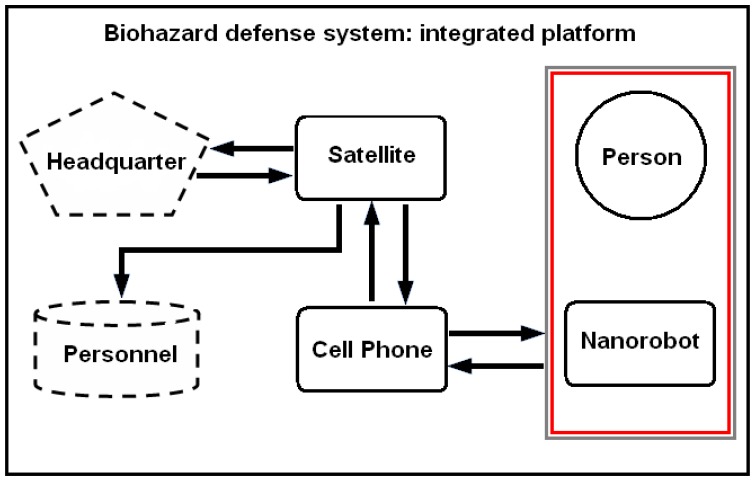
Military strategic and tactical relay satellites can use ultra high frequency for long distance epidemic monitoring and control, back tracking information from the mobile phone. Communication interface provides person identification and position, using nanorobots with PDA smart cell phone.

The application of ultra-high frequency satellite communications network can be successfully applied for nanorobot data transmission, using wireless phones for long distance communication [7,68,69,107]. The cell phone PDA (personal digital assistant) system provides also the person's identification with respective position for the moment the nanorobot detected some virus protein profile (Fig. 10).

**Figure 11.**
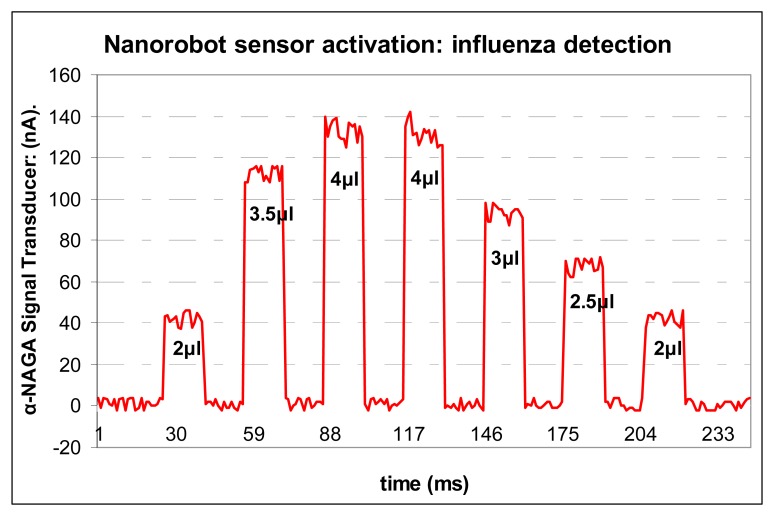
Nanobiosensor activation.

**Figure 12.**
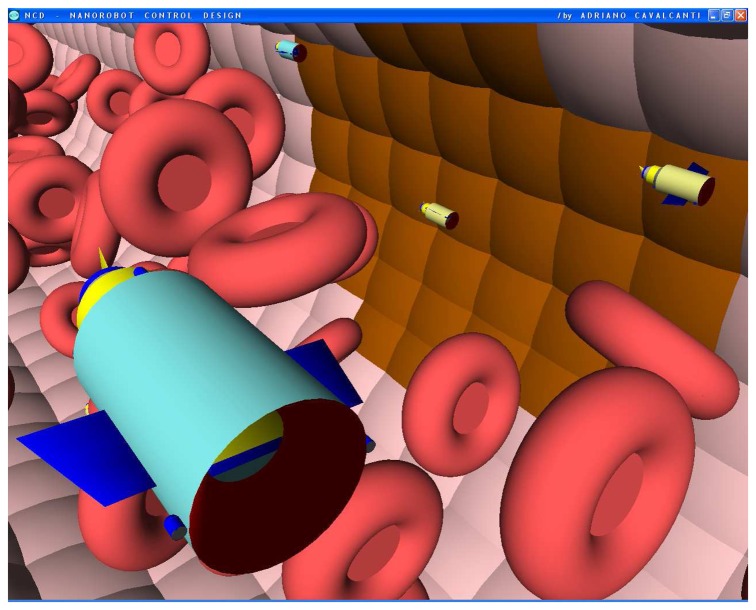
Nanorobots detecting higher concentrations of alpha-NAGA signals within the bloodstream.

Carbon nanotubes serve as ideal materials for the basis of a CMOS IC biosensor. In fact, carbon based sensor has been used successfully for *in vivo* protein detection [108]. Considering the importance of alpha-NAGA against neuroaxonal dystrophy [109], small concentrations of this protein inside body can cause some false positives. Typical concentrations of alpha-NAGA protein are less than 1 nmole/min/106 cells. Normal concentrations of alpha-NAGA in the bloodstream are in average less than 2μl. For a person infected with influenza, alpha-NAGA concentrations from blood sample increases, ranging from 1.26 to 4.63 nmole/min/106 cells [16]. Therefore, if the nanorobot's electrochemical sensor detects alpha-NAGA in low quantities or inside expected gradients, it generates a weak signal lower than 50nA. In such case the nanorobot ignores the alpha-NAGA concentration, assuming it as expected levels of bloodstream concentration. However, if the alpha-NAGA reaches concentration higher than 3μl, it produces a current flow that corresponds to the rate of antigen enzymatic reaction, which generates a current higher than 80nA (Fig. 11), hence activating the nanorobot. Every time it happens (Fig. 12), the nanorobot emits an electromagnetic signal back-propagated for the monitoring integrated platform, which records the cell phone PDA associated with the person identification [107]. This approach can enable the headquarters to automatically identify the person infected with influenza, and send an urgent SMS to multiple recipients. Therefore, the members of a same group can take the necessary action to immediately assist who was infected, avoiding any possible pandemic outbreak.

**Figure 13.**
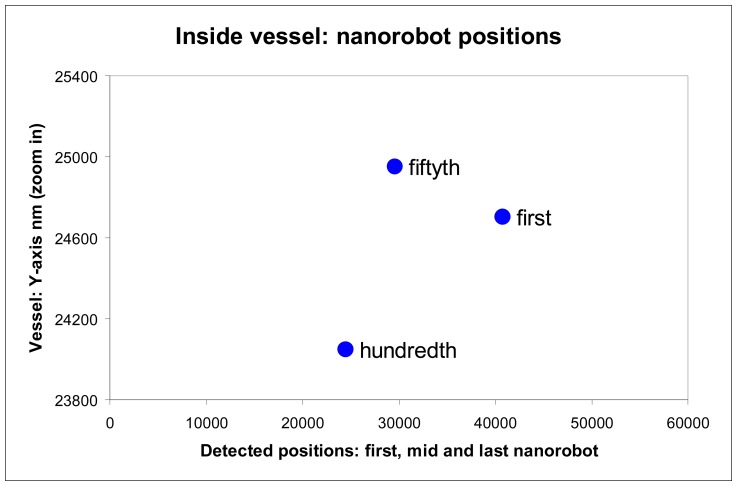
Nanorobots activation inside vessel with respective Y-X positions.

**Figure 14.**
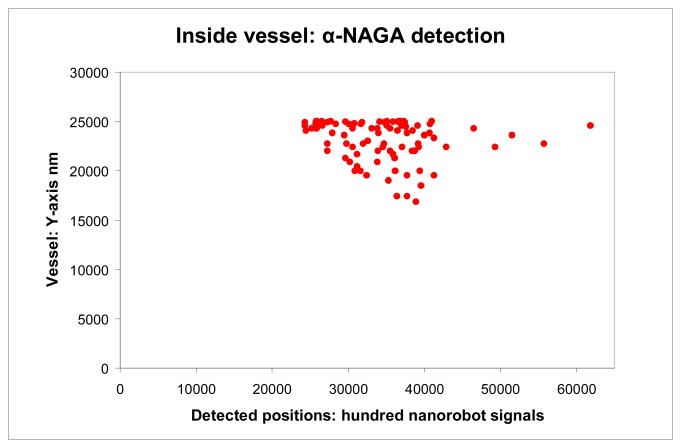
Electromagnetic back propagated signals generated from nanorobots activation.

As a threshold to avoid noise distortions and achieve a higher resolution, at least a total of 100 nanorobots must emit a higher proteomic signal transduction for a same person (Fig. 13). In such case, the system considers a strong evidence of influenza contamination. Thus, the medical nanorobot can be extremely useful to identify a patient with early development of influenza (Fig. 14).

## 11. Molecular Machine Manufacturing

Developments on nanobioelectronics and proteomics should enable fully operational nanorobots, integrated as molecular machines, for use in common medical applications [42,110-112]. In the present approach, the proposed architecture assembles as a nanoelectronic biochip integration process [5,113-116]. Progress in technology has historically shown that technical challenges can be converted to opportunities [117]. Thus, although important breakthroughs are demanded for the fully implementation of hardware to enable nanorobots, the main barriers could be successfully overcome by research and continuous development. For example, lithography has successfully enabled manufacturing of compact components comprising several nanowire layers to integrate nanoelectronics [118-120]. CMOS has enhanced miniaturization and industrial manufacturing techniques [119,121,122], which have provided ways to achieve commercialized products as nanoelectronics integrated devices. Nanosensors using DNA and CNT as innovative materials were successfully demonstrated for protein detection [112,120,123,124]. The recent implementation of high-K/metal-gate in the 45-nm silicon technology node should result in positive impact on the progress of high-K research for InSb (indium antimonide) and InGaAs (indium gallium arsenide) [119], enabling new ways to achieve smaller nano-IC packaging. In the same time, block copolymer can be viewed as a promising approach to improve manufacturing miniaturization of current nanoelectronics [122,125], even enabling complex 3D nanodevices, not previously allowed by traditional CMOS techniques. Those methods and new materials should therefore be investigated together to enable more complex nanoelectronic packaging, such as necessary for integration of nanorobots. To extend further the CMOS performance improvements found with dimensional scaling, new materials for planar MOSFETs and non-classical MOSFET structures are currently in development, which should also be considered to advance nanoelectronics and new biosensors mostly useful for nanomedicine [126,127].

## 12. Conclusions and Outlook

This work used a 3D approach to show how nanorobots can effectively improve health care and medical defense. Nanorobots should enable innovative real time protection against pandemic outbreaks. The use of nanomechatronics techniques and computational nanotechnology can help in the process of transducers investigation and in defining strategies to integrate nanorobot capabilities. A better comprehension about the requirements a nanorobot should address, in order to be successfully used for *in vivo* instrumentation, is a key issue for the fast development of medical nanorobotics. Details on current advances on nanobioelectronics were used to highlight pathways to achieve nanorobots as an integrated molecular machine for nanomedicine. Moreover, based on achievements and trends in nanotechnology, new materials, photonics, and proteomics, a new investigation methodology, using clinical data, numerical analysis and 3D simulation, has provided a nanorobot hardware architecture with real time integrated platform for practical long distance medical monitoring. This model can enable nanorobots as innovative biohazard defense technology.

In the 3D simulation, the nanorobots were able to efficiently detect alpha-NAGA signals in the bloodstream, with the integrated system retrieving information about a person infected with influenza. The model provided details on design for manufacturability, major control interface requirements, and inside body biomolecular sensing for practical development and application of nanorobots in medical prognosis.

The use of nanorobots for *in vivo* monitoring chemical parameters should significantly increase fast strategic decisions. Thus, nanorobot for medical defense means an effective way to avoid an aggressive pandemic disease to spread into an outbreak. As a direct impact, it should also help public health sectors to save lives and decrease high medical costs, enabling a real time quarantine action. An important and interesting aspect in the current development is the fact that, the similar architecture presented in terms of hardware and platform integration, can also be used to detect most types of biohazard contaminants. The research and development of nanorobots for common application in fields such as medicine and defense technology should lead us for a safer and healthier future.
